# Effectiveness and current status of multidisciplinary care for patients with chronic kidney disease in Japan: a nationwide multicenter cohort study

**DOI:** 10.1007/s10157-023-02338-w

**Published:** 2023-03-31

**Authors:** Masanori Abe, Tsuguru Hatta, Yoshihiko Imamura, Tsutomu Sakurada, Shinya Kaname

**Affiliations:** 1 The Committee of the Evaluation and Dissemination for Certified Kidney Disease Educator, Japan Kidney Association, Tokyo, Japan; 2 Division of Nephrology, Hypertension and Endocrinology, Department of Medicine, Nihon University School of Medicine, Tokyo, Japan; 3 Department of Medicine, Hatta Medical Clinic, Kyoto, Japan; 4 Department of Nephrology, Nissan Tamagawa Hospital, Tokyo, Japan; 5 Division of Nephrology and Hypertension, Department of Internal Medicine, St. Marianna University School of Medicine, Kanagawa, Japan; 6 Department of Nephrology and Rheumatology, Kyorin University School of Medicine, Tokyo, Japan

**Keywords:** Certified Kidney Disease Educator, Chronic kidney disease, Estimated glomerular filtration rate, Kidney function, Multidisciplinary care, Renal replacement therapy

## Abstract

**Background:**

Multidisciplinary care is well established in clinical practice, but its effectiveness in patients with chronic kidney disease (CKD) remains unclear. The aim of this study was to determine whether multidisciplinary care could help to avoid worsening kidney function in patients with CKD.

**Methods:**

This nationwide study had a multicenter retrospective observational design and included 3015 Japanese patients with CKD stage 3–5 who received multidisciplinary care. We assessed the annual decrease in estimated glomerular filtration rate (ΔeGFR) and urinary protein in the 12 months before and 24 months after the start of multidisciplinary care. All-cause mortality and initiation of renal replacement therapy were investigated according to baseline characteristics.

**Results:**

Most of the patients had CKD stage 3b or higher and a median eGFR of 23.5 mL/min/1.73 m^2^. The multidisciplinary care teams consisted of health care professionals from an average of four disciplines. ΔeGFR was significantly smaller at 6, 12, and 24 months after initiation of multidisciplinary care (all *P* < 0.0001), regardless of the primary cause of CKD and its stage when multidisciplinary intervention was started. Urinary protein level also decreased after initiation of multidisciplinary care. After a median follow-up of 2.9 years, 149 patients had died and 727 had started renal replacement therapy.

**Conclusion:**

Multidisciplinary care may significantly slow the decline in eGFR in patients with CKD and might be effective regardless of the primary disease, including in its earlier stages. Multidisciplinary care is recommended for patients with CKD stage 3–5.

**Trial registration:**

UMIN00004999.

**Supplementary Information:**

The online version contains supplementary material available at 10.1007/s10157-023-02338-w.

## Introduction

The number of patients with chronic kidney disease (CKD) is growing around the world. Approximately 13.3 million adults in Japan were estimated to have CKD in 2005 [1], and this number had increased to 14.8 million by 2015, potentially reflecting the aging population in Japan [2]. Accordingly, the number of patients with end-stage kidney disease starting renal replacement therapy (RRT) in Japan is increasing annually and the number of patients who are undergoing dialysis therapy now exceeds 340,000 [3]. The prevalence of dialysis in Japan is 2682 per million population, which is the second highest worldwide after Taiwan [4]. There are numerous risk factors for progressive CKD, including hypertension, diabetes, and advancing age, which result in worsening kidney function that can lead to end-stage kidney disease and cardiovascular disease (CVD). CKD is an internationally recognized public health problem because of its epidemiological features, high mortality rate, and considerable medical costs [5]. Therefore, important treatment goals in patients with CKD are slowing of disease progression, minimizing complications, and improving quality of life.

The multidisciplinary care model encompasses a range of disciplines with different but complementary skills, knowledge, and experience and aims to improve health care and achieve optimal outcomes in terms of the physical and psychosocial needs of patients [6]. However, there is still a need to improve the standard care for patients with CKD in clinical practice. The Certified Kidney Disease Educator (CKDE) system was established in Japan by the Japan Kidney Association (JKA) in 2017 with the aims of preventing progression of CKD and improving and maintaining patients’ quality of life. Nurses, registered dietitians, and pharmacists who meet certain requirements are eligible for qualification as a CKDE. All CKDEs have acquired the basic skills for management of patients with CKD, including guidance on lifestyle modification, dietary counseling, and medical therapy according to stage of CKD. Thus, CKDEs play an important role in multidisciplinary care. By 2022, there were 1935 CKDEs in Japan, and multidisciplinary care of patients with CKD by board-certified nephrologists and CKDEs has become widespread. However, only a limited number of studies in Japan have investigated the association between multidisciplinary care for patients with CKD and kidney function, and these studies involved small numbers of patients from single centers [7, 8]. In this multicenter cohort study, we investigated the current status of multidisciplinary care for patients with CKD and whether multidisciplinary care can help to avoid worsening of kidney function in patients with CKD.

## Methods

### Study design and participants

This nationwide study was designed as a multicenter retrospective observational cohort study involving approximately 3000 Japanese patients who were enrolled at 24 selected medical institutions in Japan. Patients with CKD who received continuous multidisciplinary care between January 2015 and December 2020 and had kidney function data available for the 12 months before and the 24 months after receiving multidisciplinary care were included.

The following exclusion criteria were applied: age younger than 20 years; estimated glomerular filtration rate (eGFR) ≥ 60 mL/min/1.73 m^2^; active malignant disease; transplant recipient status; history of long-term dialysis; and missing data on age, sex, or kidney function. The primary efficacy endpoint was the annual decline in eGFR (ΔeGFR) between 12 months before and 24 months after the start of multidisciplinary intervention. Secondary endpoints were the annual change in the urinary protein level between 12 months before and 24 months after the start of multidisciplinary intervention and the composite outcome of all-cause mortality and initiation of RRT until the end of 2021.

The study was approved by the ethics committee of Nihon University Itabashi Hospital and conducted in accordance with the Declaration of Helsinki, Japanese privacy protection laws, and the Ethical Guidelines for Medical and Health Research Involving Human Subjects published by the Ministry of Education, Culture, Sports, Science and Technology and the Ministry of Health, Labour and Welfare in 2015. The need for informed consent was waived due to the use of de-identified data. Information in this study was disclosed to subjects in an opt-out format. The study is registered in the University Hospital Medical Information Network (UMIN000049995).

### Multidisciplinary care

Multidisciplinary care was defined as follows: (1) a care team comprising nephrologists and professionals from other disciplines, including nurses, registered dietitians, pharmacists, physical therapists, social workers, clinical engineers, and clinical laboratory technicians; and (2) an operational model of multidisciplinary care, whereby patients with CKD were managed medically, received patient education, and were encouraged to make lifestyle modifications according to the stage of CKD. The quality of the educational content provided was maintained in accordance with the recommendations of the Japanese Society of Nephrology, Japanese Society for Dialysis Therapy, Japan Society for Transplant, and Japanese Society for Clinical Renal Transplantation or the CKD Teaching Guidebook for Certified Kidney Disease Educators by the JKA [9, 10].

### Data collection

Data were collected on patient demographics and clinical characteristics, including age, sex, history of CVD, primary etiology of CKD, body mass index (BMI), hemoglobin, serum albumin, blood urea nitrogen, creatinine (Cr), eGFR, urinary protein, and glycated hemoglobin (for patients with diabetes) at the time when multidisciplinary care intervention was initiated (baseline). CVD was defined as coronary artery disease, ischemic stroke, hemorrhagic stroke, and limb amputation. The eGFR was calculated according to the following formula for Japanese patients: eGFR (mL/min/1.73 m^2^) = 194 × serum Cr^− 1.094^ × age^−0.287^(× 0.739 for women) [11]. Urinary protein was calculated as the urinary protein to creatinine ratio (UPCR). The eGFR and UPCR values at 12 months before the intervention and at 6, 12, and 24 months after the start of the intervention were obtained. Information on the method and setting of intervention (outpatient or inpatient), duration of intervention (number of visits for intervention for outpatients or hospitalization days for inpatients), and type and number of staff was collected. The composite outcome of all-cause mortality and initiation of RRT was assessed using dates of death and initiation of RRT or the end of 2021 was reached, whichever came first. The type of RRT (i.e., hemodialysis, peritoneal dialysis, or kidney transplantation) was recorded.

### Statistical analysis

Data are reported as the number and proportion, mean ± standard deviation, or median [interquartile range]. Categorical variables were examined using the chi-squared test, and continuous variables were compared using the *t* test. Three or more groups were compared using repeated-measures analysis of variance with Tukey’s honestly significant difference test or the Kruskal–Wallis test, as appropriate. The associations between the number of multidisciplinary care team members and the number of interventions by the multidisciplinary care team, and the mean ΔeGFR and the % changes in UPCR were analyzed using Spearman's rank correlation coefficient. Incidence of all-cause death and incidence of initiation of RRT are presented as the number of events per 1000 person-years. For survival analysis of the composite outcome, the patients were divided into two groups according to diabetes mellitus (DM) status and four groups according to CKD stage (G3a, G3b, G4, or G5) at baseline. The composite outcome was estimated using the Kaplan–Meier method and compared between groups using the log-rank test. A univariate analysis was performed according to eGFR stage, and multivariate survival analyses were performed using Cox proportional hazards models adjusted for confounders to examine associations between baseline CKD stage and the composite outcome during 6 years of follow-up. Model 1 was used to calculate the hazard ratios adjusted for basic characteristics, including age, sex, history of CVD, and DM status. Model 2 was the same as model 1 but was further adjusted for BMI, hemoglobin, serum albumin, and UPCR levels. A univariate analysis was performed according to DM status, and multivariate survival analyses using Cox proportional hazards models adjusted for confounding factors were performed to examine DM status and the composite outcome. Model 1 was used to calculate the hazard ratios adjusted for basic factors, including age, sex, and history of CVD, and model 2 was adjusted for BMI, hemoglobin, serum albumin, eGFR, and UPCR levels in addition to the factors included in model 1. The results from the models are expressed as hazard ratios (HRs) with 95% confidence intervals (CIs) and P-values. Multivariate survival analyses were performed using Cox proportional hazards models adjusted for confounders to examine associations between the number of multidisciplinary care team members and the number of multidisciplinary care team interventions and composite outcomes. Moreover, to discover which factors and specialty compositions within the multidisciplinary care team are advantageous for the composite endpoint, we estimated the HRs and compared them between the group with each specialist member present and the group without as the reference group. For the regression analyses, imputation of missing data was performed by conventional methods, as appropriate. All analyses were performed using JMP^®^ version 13.0 (SAS Institute Inc., Cary, NC, USA). A *P* value < 0.05 was considered statistically significant.

## Results

### Patient characteristics at time of initiation of multidisciplinary care

Of 3146 patients registered during the study period, 131 were excluded (CKD stage 1 or 2, *n* = 118; no baseline kidney function data, *n* = 13), leaving 3015 patients for inclusion in the analysis. The patients’ background characteristics are shown in Table 1. Mean age was 70.5 ± 11.6 years and 74.2% were male. In terms of disease severity, median eGFR was 23.5 [15.1–34.4] mL/min/1.73 m^2^ and median UPCR was 1.13 [0.24–3.1] g/gCr. CKD was stage 4 in 1248 patients (41.4%), stage 3b in 761 (25.2%), and stage 5 in 726 (24.1%). Diabetic nephropathy was the most common primary cause of CKD, followed by hypertension and glomerulonephritis.

**Table 1:** Baseline characteristics of the patients

Variable	
Patients, *n* (% male)	3015 (74.2)
Age, years	70.5 ± 11.6
Body mass index	24.2 ± 4.3
Serum creatinine, mg/dL	2.08 [1.48–3.14]
eGFR, mL/min/1.73 m^2^	23.5 [15.1–34.4]
Blood urea nitrogen, mg/dL	32 [23–45]
Hemoglobin, g/dL	11.7 ± 1.9
Serum albumin, g/dL	3.7 ± 0.5
Urinary protein, g/gCr	1.13 [0.24–3.1]
Comorbid CVD, *n* (%)	885 (29.4)
HbA_1c_ (in DM patients), %	6.4 ± 1.0
CKD stage, *n* (%)	
3 (3a + 3b)	1041 (34.5)
3a	280 (9.3)
3b	761 (25.2)
4	1248 (41.4)
5	726 (24.1)
Primary cause of CKD, *n* (%)	
Diabetes	1321 (43.8)
Hypertension	894 (29.7)
Glomerulonephritis	384 (12.7)
PCKD	88 (2.9)
Other	328 (10.9)

Data are shown as the number (percentage), mean ± standard deviation, or median [interquartile range]

*Cr* creatinine, *CKD* chronic kidney disease, *CVD* cardiovascular disease, *DM* diabetes mellitus, *eGFR* estimated glomerular filtration rate, *HbA*_*1c*_ glycated hemoglobin, *PCKD* polycystic kidney disease

### Interventions implemented by the multidisciplinary care team

Details of the interventions implemented by the multidisciplinary care team are shown in Table 2. Intervention was provided in an inpatient setting for more than half of the patients and on an outpatient basis for the remainder. The majority of the multidisciplinary team members were registered dieticians (90.4%), followed by nurses (86.2%), pharmacists (62.3%), and physical therapists (25.9%). The mean number of multidisciplinary care team members was four; 33.7% of the patients received intervention by five team members and 29.2% by four team members.

**Table 2:** Characteristics of the multidisciplinary care team and interventions

Variable	
Place of intervention, *n* (%)	
Outpatient	1246 (41.3)
Inpatient	1769 (58.7)
Number of interventions	
Outpatient setting, *n*	4 [1–11]
Inpatient setting, *n*	7 [6–12]
Professional makeup of MDC team, *n* (%)	
Nurses	2600 (86.2)
Registered dieticians	2726 (90.4)
Pharmacists	1878 (62.3)
Physical therapists	781 (25.9)
Clinical laboratory technicians	178 (5.9)
Social workers	72 (2.3)
Other professionals	31 (1.0)
Number of MDC team members, *n* (%)	4 [3–5]
2	700 (23.2)
3	416 (13.8)
4	882 (29.2)
5	994 (33.0)
6	23 (0.8)

Data are shown as the number (percentage), mean ± standard deviation, or median [interquartile range]

*MDC* multidisciplinary care

### ΔeGFR before and after multidisciplinary care

**Fig. 1:**
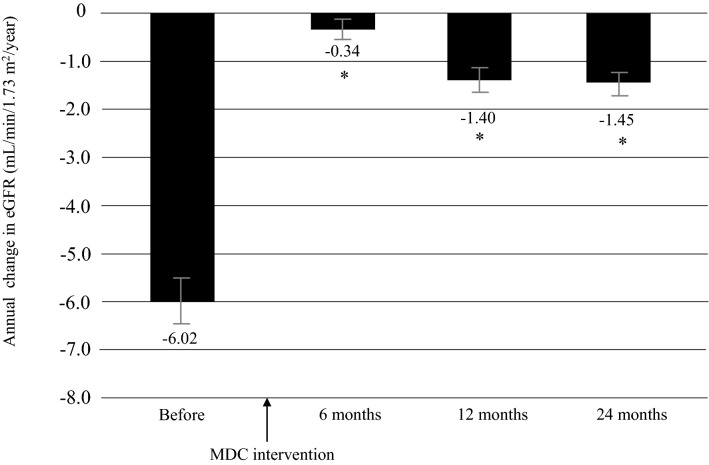
Annual changes in eGFR decline (ΔeGFR) in the 12 months before and 24 months after initiation of multidisciplinary care in all patients. Data are shown as the mean. Bars indicate the 95% confidence interval. *P < 0.0001 vs. before start of MDC. eGFR estimated glomerular filtration rate, MDC multidisciplinary care

**Fig. 2:**
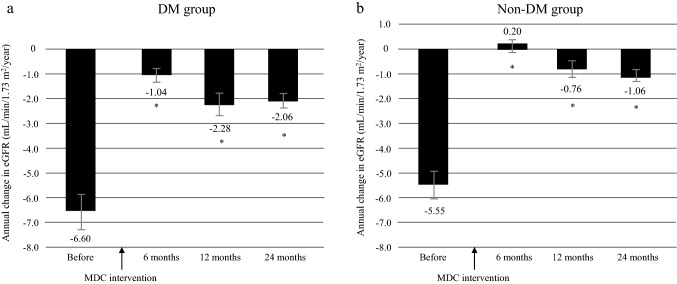
Annual changes in eGFR decline (ΔeGFR) in the 12 months before and 24 months after initiation of multidisciplinary care according to DM status. **a** DM group, **b** non-DM group. *P < 0.0001 vs. before start of MDC. Data are shown as the mean. Bars indicate the 95% confidence interval. DM diabetes mellitus, eGFR estimated glomerular filtration rate, MDC multidisciplinary care

The mean annual decline in eGFR (ΔeGFR) was − 6.0 ± 9.0 before multidisciplinary intervention and − 0.34 ± 5.78 at 6 months, − 1.40 ± 6.82 at 12 months, and − 1.45 ± 4.04 at 24 months after intervention (all *P* < 0.0001; Fig. 1). Furthermore, in the DM group, mean ΔeGFR was − 6.60 ± 9.5 before intervention and − 1.04 ± 5.92 at 6 months, − 2.28 ± 7.39 at 12 months, and − 2.06 ± 4.50 at 24 months after intervention (all *P* < 0.0001; Fig. 2a); the respective values in the non-DM group were − 5.55 ± 8.56, 0.20 ± 5.61, − 0.76 ± 6.29, and − 1.06 ± 3.66 (all *P* < 0.0001; Fig. 2b).

**Fig. 3:**
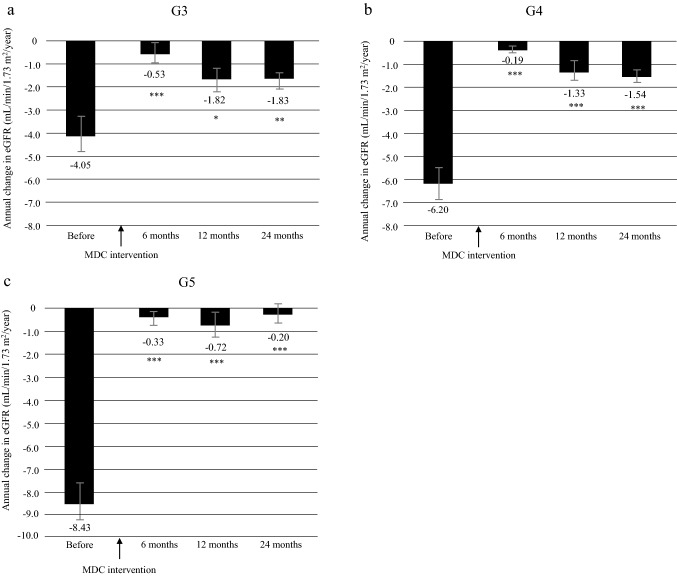
Annual changes in decline of eGFR (ΔeGFR) in the 12 months before and 24 months after initiation of multidisciplinary care according to CKD stage at the time of initiation of MDC. **a** CKD stage G3, **b** CKD stage G4, **c** CKD stage G5. ***P < 0.0001, **P < 0.001, *P < 0.01 vs. before start of MDC. Data are shown as the mean. Bars indicates the 95% confidence interval. CKD chronic kidney disease, eGFR estimated glomerular filtration rate, MDC multidisciplinary care

In patients with CKD stage 3, mean ΔeGFR was − 4.05 ± 9.19 before intervention and − 0.53 ± 6.84 at 6 months, − 1.82 ± 7.43 at 12 months, and − 1.83 ± 4.21 at 24 months after intervention; the difference was significant at all assessment points after intervention (Fig. 3a). When the patients with CKD stage 3 were divided into G3a and G3b subgroups, the difference in mean ΔeGFR was significant only for stage G3b (Supplementary Fig. 1). For patients with CKD stage 4, mean ΔeGFR was − 6.20 ± 8.35 before intervention and − 0.19 ± 5.01 at 6 months, − 1.33 ± 6.14 at 12 months, and − 1.54 ± 3.66 at 24 months after intervention (all *P* < 0.0001; Fig. 3b); the respective values in patients with CKD stage 5 were − 8.43 ± 9.13, − 0.33 ± 5.42, − 0.72 ± 6.98, and − 0.20 ± 4.36 (all *P* < 0.0001; Fig. 3c).

There was no significant correlation between the mean ΔeGFR and the number of multidisciplinary care team members, but there was a significant correlation between the mean ΔeGFR and number of interventions by the multidisciplinary care team at all time points (all *P* < 0.05; Supplementary Table 1).

### Changes in proteinuria after multidisciplinary intervention

**Fig. 4:**
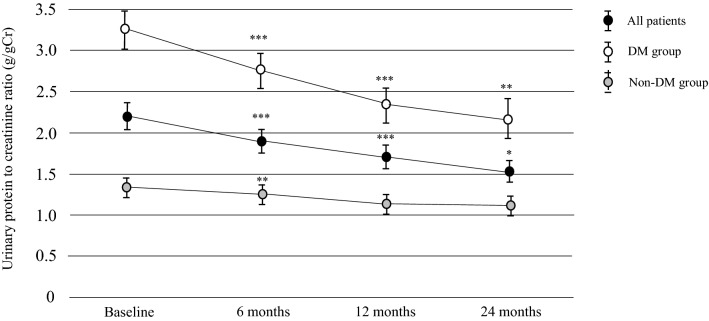
Changes in urinary protein levels between the time of initiation of MDC and 24 months after initiation of multidisciplinary care according to DM status. ***P < 0.0001, **P < 0.001, *P < 0.05 vs. baseline. Data are shown as the mean. Bars indicates the 95% confidence interval. DM diabetes mellitus

Median UPCR decreased significantly from 1.13 [0.24–3.10] g/gCr at baseline to 0.96 [0.23–2.63] g/gCr at 6 months (*P* < 0.0001), 0.82 [0.21–2.30] g/gCr at 12 months (*P* < 0.0001), and 0.78 [0.19–2.07] g/gCr at 24 months (*P* = 0.019) after intervention in all patients. There was a significant decrease in UPCR at all measurement times after intervention in the DM group but only at 6 months in the non-DM group (*P* = 0.0003) (Fig. 4).

There was a significant correlation between the % changes in UPCR and the number of multidisciplinary care team members at 12 and 24 months after intervention, but no significant correlation between the % changes in UPCR and the number of interventions by the multidisciplinary care team at all time points (Supplementary Table 2).

### Outcomes

The median observation period was 35 [20–50] months, during which 149 patients (4.9%) died, 747 (24.8%) started RRT, and 66 (2.2%) were lost to follow-up. RRT consisted of hemodialysis in 618 patients (82.7%), peritoneal dialysis in 66 (8.8%), and renal transplantation in 25 (3.5%).

**Fig. 5:**
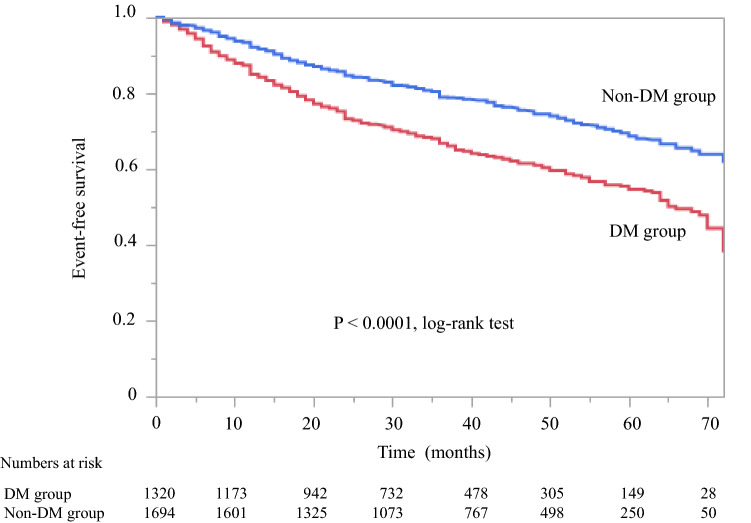
Kaplan–Meier curves for the incidence of all-cause death and initiation of renal replacement therapy in Japanese patients with CKD according to DM status. CKD chronic kidney disease, DM diabetes mellitus

The characteristics and outcomes according to DM status are shown in Table 3. Patients in the DM group were more likely to be male, have comorbid CVD, be younger, and to have higher BMI and UPCR and lower eGFR and serum albumin levels. Kaplan–Meier analysis for the composite endpoint (all-cause mortality and initiation of RRT) revealed a significant difference between the DM and non-DM groups (*P* < 0.0001, log-rank test; Fig. 5). Compared with the non-DM (reference) group, the DM group had a significant higher unadjusted HR for all-cause mortality and initiation of RRT (1.74, 95% CI 1.53–1.99, *P* < 0.0001). After adjustment for background factors, including age, sex, and history of CVD, the HR in the DM group was 1.68 (95% CI 1.47–1.93, *P* < 0.0001). After further adjustment for background factors and laboratory data, including BMI, hemoglobin, serum albumin, eGFR, and UPCR level at baseline, the DM group had a significantly higher HR (1.28, 95% CI 1.09–1.51, *P* < 0.0001) (Table 4). Kaplan–Meier analysis revealed a significant difference in all-cause mortality between the DM and non-DM groups (*P* = 0.031, log-rank test; Supplementary Fig. 2). After adjustment for background factors, including age, sex, and history of CVD, the HR in the DM group compared with the non-DM group (reference) was 1.49 (95% CI 1.08–2.06). After further adjustment for background factors and laboratory data, including BMI, hemoglobin, serum albumin, eGFR, and UPCR level at baseline, the HR was significantly higher in the DM group (1.49, 95% CI 1.01–2.19, *P* = 0.044) (Supplementary Table 3).

**Table 3:** Baseline characteristics according to DM status

Variable	DM	Non-DM	*P* value
Patients, *n*	1321	1694	–
Male sex, %	78.1	71.2	< 0.0001
Age, years	69.4 ± 11.4	71.4 ± 11.7	< 0.0001
Body mass index	24.9 ± 4.7	23.7 ± 3.9	< 0.0001
Serum creatinine, mg/dL	2.65 ± 1.5	2.39 ± 1.4	< 0.0001
eGFR, mL/min/1.73 m^2^	24.4 ± 12.5	26.5 ± 12.9	< 0.0001
Blood urea nitrogen, mg/dL	37.0 ± 17.5	35.7 ± 17.5	0.040
Hemoglobin, g/dL	11.5 ± 1.9	11.8 ± 1.9	< 0.0001
Serum albumin, g/dL	3.6 ± 0.6	3.8 ± 0.5	< 0.0001
Urinary protein, g/gCr	2.20 [0.57–4.90]	0.62 [0.15–1.79]	< 0.0001
Comorbid CVD, *n* (%)	436 (33.0)	449 (26.5)	0.0004
HbA_1c_ (in DM patients), %	6.6 ± 1.1	–	–
CKD stage, *n* (%)			0.0005
3 (3a + 3b)	406 (30.7)	635 (37.5)	
3a	106 (8.0)	174 (10.3)	
3b	300 (22.7)	461 (27.2)	
4	561 (42.5)	687 (40.6)	
5	354 (26.8)	372 (21.9)	
Observation period, months	33 [17–48]	36 [22–52]	< 0.0001
All-cause death, *n* (%)	75 (5.7)	75 (4.4)	0.132
All-cause death, per 1000 person-years	20.3	14.2	0.031
Initiation of RRT, *n* (%)	416 (31.5)	331 (19.5)	< 0.0001
Initiation of RRT, per 1000 person-years	113	62.8	< 0.0001

Data are shown as the number, percentage, mean ± standard deviation, or median [interquartile range]

*Cr* creatinine, *CKD* chronic kidney disease, *CVD* cardiovascular disease, *DM* diabetes mellitus, *eGFR* estimated glomerular filtration rate, *RRT* renal replacement therapy

**Table 4:** All-cause mortality and initiation of renal replacement therapy according to DM status in Cox proportional hazards models adjusted for confounding factors in Japanese patients with CKD

Group	Unadjusted			Model 1			Model 2		
	HR	95% CI	*P* value	HR	95% CI	*P* value	HR	95% CI	*P* value
Non-DM	1.00	Reference	–	1.00	Reference	–	1.00	Reference	–
DM	1.74	1.53–1.99	< 0.0001	1.68	1.47–1.93	< 0.0001	1.28	1.09–1.51	< 0.0001

Model 1 was adjusted for basic factors, including age, sex, and history of cardiovascular disease, and model 2 was adjusted in the same way as model 1 but with additional adjustment for body mass index, hemoglobin, serum albumin, estimated glomerular filtration rate, and urinary protein level at baseline

*CI* confidence interval, *CKD* chronic kidney disease, *DM* diabetes mellitus, *eGFR* estimated glomerular filtration rate, *HR* hazard ratio

**Fig. 6:**
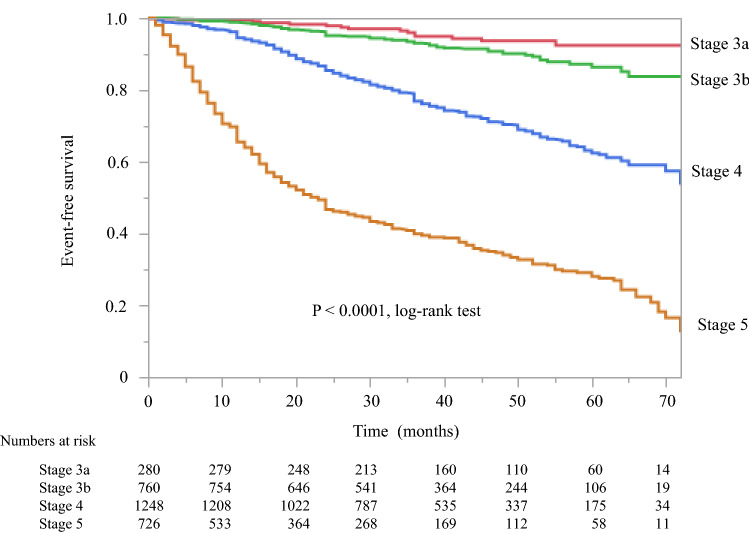
Kaplan–Meier curves for the incidence of all-cause death and initiation of renal replacement therapy initiation at baseline in Japanese patients with CKD according to CKD stage. CKD chronic kidney disease

Patient characteristics and outcomes according to CKD stage are shown in Table 5. BMI, hemoglobin, the serum albumin level, and the glycated hemoglobin value (for patients with diabetes) decreased while the UPCR level increased with progression though the stages of CKD. All-cause mortality and the RRT initiation rate were dependent on the disease stage. Significant differences (all *P* < 0.0001, log-rank test) were found in the composite endpoint (all-cause death or RRT initiation) according to CKD stage at baseline in Japanese patients with CKD (Fig. 6). Kaplan–Meier analysis revealed that all-cause mortality varied significantly depending on the CKD stage at baseline (*P* = 0.0009, log-rank test; Supplementary Fig. 2). After adjustment for basic factors, including age, sex, history of CVD, and DM status, the HRs in the G3b, G4, and G5 groups when compared with the G3a (reference) group were 2.43 (95% CI 1.04–7.08), 2.49 (95% CI 1.11–7.17), and 3.77 (95% CI 1.61–11.0), respectively. However, after adjustment for basic factors and laboratory data, including BMI, hemoglobin, serum albumin, and UPCR level, only the G5 group had a significantly higher HR (3.03, CI 1.01–9.11, *P* = 0.048; Supplementary Table 4).

**Table 5:** Comparison of patient characteristics and outcomes according to CKD stage at baseline

Variable	Stage 3a	Stage 3b	Stage 4	Stage 5	*P* value
Patients, *n*	280	761	1248	726	–
Male sex, %	77.5	77.9	74.4	68.9	0.0005
Age, years	65.7 ± 12.1	70.3 ± 10.9	71.7 ± 11.6	70.6 ± 11.8	< 0.0001
Body mass index, kg/m^2^	24.9 ± 4.3	24.2 ± 4.1	24.3 ± 4.4	23.7 ± 4.3	0.001
Serum creatinine, mg/dL	1.07 ± 0.14	1.44 ± 0.23	2.31 ± 0.53	4.44 ± 1.37	< 0.0001
eGFR, mL/min/1.73 m^2^	51.0 ± 3.9	36.4 ± 4.2	21.8 ± 4.4	10.8 ± 2.7	< 0.0001
Blood urea nitrogen, mg/dL	18 [15–21]	23 [20–27]	34 [28–42]	53 [44–64]	< 0.0001
Hemoglobin, g/dL	13.7 ± 1.6	12.6 ± 1.8	11.4 ± 1.6	10.4 ± 1.5	< 0.0001
Serum albumin, g/dL	3.9 ± 0.5	3.9 ± 0.5	3.7 ± 0.5	3.6 ± 0.5	< 0.0001
Urinary protein, g/gCr	0.33 [0.08–1.32]	0.33 [0.09–1.43]	1.20 [0.32–3.21]	2.59 [1.26–4.98]	< 0.0001
Comorbid CVD, *n* (%)	71 (25.4)	211 (27.8)	394 (31.6)	209 (28.8)	0.0002
HbA1c (in DM patients), %	6.7 ± 1.1	6.5 ± 1.1	6.4 ± 1.0	6.2 ± 0.9	< 0.0001
Primary cause of CKD, *n* (%)					0.0024
Diabetes	106 (37.9)	299 (39.3)	561 (45.0)	354 (48.8)	
Hypertension	97 (34.6)	245 (32.2)	381 (30.5)	171 (23.6)	
Glomerulonephritis	39 (13.9)	97 (12.8)	146 (11.7)	102 (14.0)	
PCKD	6 (2.1)	24 (3.2)	35 (2.8)	23 (3.2)	
Other	32 (11.4)	95 (12.5)	125 (10.0)	76 (10.4)	
Observation period, months	44 [30–56]	40 [28–53]	36 [23–51]	29 [9–37]	< 0.0001
All-cause mortality, *n* (%)	5 (1.8)	37 (4.9)	69 (5.5)	39 (5.4)	< 0.0001
All-cause mortality, per 1000 person-years	5.0	14.6	17.8	25.4	< 0.0001
RRT initiation, *n* (%)	9 (3.2)	30 (4.0)	268 (21.5)	440 (60.6)	< 0.0001
RRT initiation, per 1000 person-years	8.9	11.8	69.1	278	< 0.0001

Data are shown as the number (percentage), mean ± standard deviation, or median [interquartile range]

*Cr* creatinine, *CKD* chronic kidney disease, *CVD* cardiovascular disease, *DM* diabetes mellitus, *eGFR* estimated glomerular filtration rate, *HbA*_*1c*_ glycated hemoglobin, *PCKD* polycystic kidney disease, *RRT* renal replacement therapy

**Fig. 7:**
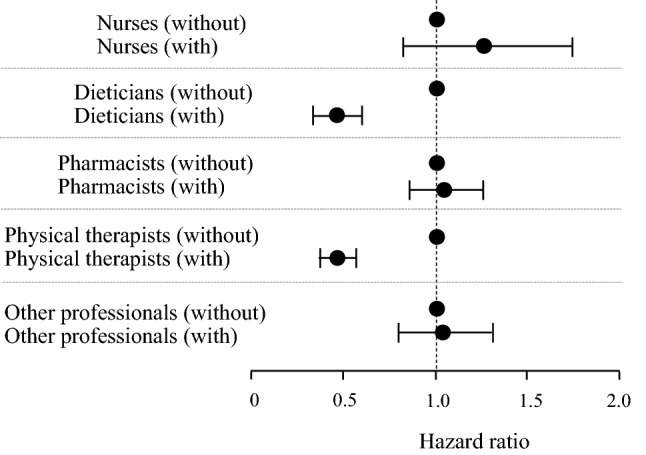
Association between specialty composition of the multidisciplinary care team and composite endpoint stratified by with or without the presence of each professional on the team. Circles indicate the adjusted hazard ratio (HR) for all-cause mortality and initiation of renal replacement therapy. Error bars indicate 95% confidence intervals (CI). The HR for the composite endpoint (95% CI) was derived from Cox proportional hazards models adjusted for all covariate values, including age, sex, history of cardiovascular disease, the presence or absence of diabetes, body mass index, hemoglobin, serum albumin, and urinary protein levels at baseline

There was a significant association between the number of multidisciplinary care team members and the composite endpoint. The HR decreased significantly with increasing numbers of multidisciplinary care team members. Also, there was a significant association between the number of interventions by the multidisciplinary care team and the composite endpoint; that is, the prognosis of the composite outcome improved as the number of interventions increased (Table 6). When we compared composite endpoints according to the specialty composition of the multidisciplinary care team, there were significantly lower HRs when registered dietitians (HR 0.47, 95% CI 0.35–0.63, *P* < 0.0001) and physical therapists (HR 0.39, 95% CI 0.31–0.48, *P* < 0.0001) were included in the multidisciplinary care team (Fig. 7).

**Table 6:** Cox proportional hazard ratios of the associations of the number of multidisciplinary care team members and the number of interventions by the multidisciplinary care team with the composite endpoint

Variables	HR	95% CI	*P* value
Number of MDC team members (increase by 1)	0.85	0.80–0.89	< 0.0001
Number of interventions by MDC team (increase by 1)	0.97	0.96–0.98	< 0.0001

*CI* confidence interval, *MDC* multidisciplinary care, *HR* hazard ratio

Presence of diabetes, being a male, history of CVD, hemoglobin, eGFR, and UPCR levels at baseline and interventions by registered dieticians and physical therapists were all identified as independent predictors of the composite outcome using multivariate Cox proportional hazard regression analysis (Table 7).

**Table 7:** The multidisciplinary care team’s multivariate Cox proportional hazard ratios of the variables connected to the composite endpoint

Variables	HR	95%CI	*P* value
Age (increase by 1 year)	0.99	0.97–1.01	0.095
Sex (male)	1.25	1.06–1.48	0.009
Diabetes (yes)	1.34	1.14–1.58	0.0003
Comorbid CVD (yes)	1.30	1.13–1.49	0.0002
Body mass index (increase by 1 kg/m^2^)	0.98	0.95–1.00	0.063
Hemoglobin (increase by 1 g/dL)	0.90	0.86–0.95	0.0002
Albumin (increase by 1 g/dL)	0.91	0.77–1.07	0.275
Baseline eGFR (increase by 1 ml/min/1.73m^2^)	0.91	0.90–0.92	< 0.0001
Baseline urinary protein (increase by 1 g/gCr)	1.08	1.05–1.11	< 0.0001
Nurses (yes)	0.89	0.55–1.42	0.617
Dieticians (yes)	0.49	0.36–0.66	0.035
Pharmacists (yes)	1.07	0.92–1.27	0.361
Physical therapists (yes)	0.46	0.22–0.93	0.017
Other professionals (yes)	0.91	0.62–1.33	0.651

*CI* confidence interval, *Cr* creatinine, *CVD* cardiovascular disease, *eGFR* estimated glomerular filtration rate, *HR* hazard ratio

## Discussion

Our nationwide cohort study included 3015 individuals from 24 facilities in Japan, 22 (91.7%) of which employ CKDEs and these 22 facilities provided intervention to 98.2% of all participating patients. Thus, the major strengths of this study are its large sample size recruited from multiple centers. Moreover, the observation period was relatively long, and a comparatively high number of elderly patients were included. Although the mean age of patients in the previous studies was younger than 70 years, our mean age was 70.5 years, reflecting our aging CKD population in Japan [12]. This study is the first to indicate that multidisciplinary care of CKD in Japan may be able to prevent worsening kidney function regardless of the underlying etiology. Multidisciplinary care was effective for patients with CKD regardless of whether they had DM. Furthermore, multidisciplinary care might be effective in the earlier stages of CKD. A multidisciplinary care team should include a nephrologist, nurse, and professionals from other fields and is recommended for patients with CKD stage 3–5. Our results suggest that the greater the number of professionals in a multidisciplinary care team, especially registered dietitians and physical therapists, the greater the number of interventions provided, which likely improves prognosis. Moreover, Japanese patients with CKD have an overwhelmingly higher rate of initiation of RRT than of mortality. The incidence of all-cause mortality in our patients with stage 3–5 CKD increased as eGFR declined but at a very low rate at all CKD stages under multidisciplinary care.

In addition to treatment and management of CKD, various lifestyle adjustments and self-management behaviors are required from the early stage of CKD through to the time of initiation and maintenance of RRT. Patients with CKD require holistic care and support, including dietary modification, maintenance and improvement of medication adherence, self-monitoring, early detection of complications, and the financial resources needed to continue treatment. Such support cannot be provided by medical staff alone and must be carried out by a medical team consisting of multiple professionals. To achieve good outcomes, multidisciplinary care teams that include nephrologists, nurses, registered dietitians, pharmacists, physical therapists, occupational therapists, and medical social workers should be involved and have shared goals for individual patients.

Multidisciplinary care has been shown to decrease all-cause mortality, reduce the need for temporary catheterization for dialysis, and decrease the hospitalization rate in patients with CKD [13–16]. In contrast, some studies have not identified significant differences in these variables according to whether patients receive multidisciplinary care [17–19]. However, a meta-analysis revealed that multidisciplinary care could decrease all-cause mortality in patients with CKD, reduce the need for temporary catheterization in patients receiving dialysis, and decrease the hospitalization rate, but only in patients with stage 4–5 disease [12]. Moreover, the CKD-JAC study found that all-cause mortality and cardiovascular event rates were lower in Japanese patients with CKD who are under the care of a nephrologist than in their Western counterparts [20–22]. The lower mortality rate in our study is consistent with the findings of the previous studies. It is thought that Japanese patients with CKD who are under the care of a nephrologist with strict management of blood pressure, metabolism, and blood glucose are at much lower risk of cardiovascular events and death than patients with CKD in Western countries, although racial differences may affect the risk [20]. Further research is needed to determine whether clinical outcomes are better in patients who receive multidisciplinary care than in those who are cared for by nephrologists alone.

The composition of the participating multidisciplinary care teams varied greatly from facility to facility in this study. It has been reported that the ideal multidisciplinary care model for patients with CKD consists of a nurse, dietician, pharmacist, and social worker in addition to a nephrologist [23]. Although some studies have found no significant difference in all-cause mortality between multidisciplinary care and non-multidisciplinary care when the multidisciplinary team included a nephrologist and a nurse [17, 19], other studies have demonstrated a significant difference in all-cause mortality when the multidisciplinary team included a nephrologist, nurse, dietician, and pharmacist [15, 16]. A meta-analysis found no significant difference in all-cause mortality when the team included a nephrologist and a nurse [12]; however, all-cause mortality was lower if the team included a nephrologist, nurse, and health care professionals from other disciplines. The present study found that addition of a nurse or dietician compared to a nephrologist alone significantly slowed the decline in eGFR. Furthermore, recent studies have identified that a higher physical activity level can slow the decline in kidney function in patients with CKD [24–27]. A guideline for exercise therapy in patients with pre-dialysis CKD and those on dialysis has been published by the Japanese Society of Renal Rehabilitation [28]. Some of the facilities in our cohort include physical therapists in their multidisciplinary care teams. Further investigations are needed to determine which and how many health care professionals are required in a multidisciplinary care team to achieve the best outcomes.

In the aforementioned meta-analysis, there was no significant difference in the all-cause mortality or hospitalization rate according to whether multidisciplinary care was received in patients with CKD stage 1–5; however, multidisciplinary care decreased both all-cause mortality and the hospitalization rate in patients with CKD stage 4–5 [12]. It is known that all-cause mortality and hospitalization rates are associated with the stage of CKD, so patients with advanced CKD (stage 4–5) would have a higher rate of cardiovascular complications and higher risk of death and hospitalization because of decreasing kidney function. Therefore, the effect of multidisciplinary care on all-cause mortality is more difficult to demonstrate in short-term studies of patients with earlier stages of CKD, which may last 1–3 years, than in those with CKD stage 4–5, in whom the effect of multidisciplinary care would be more marked. Referral to a nephrologist is recommended for patients with CKD who reach stage 4 according to the Kidney Disease: Improving Global Outcomes (KDIGO) guidelines and for patients who reach stage 3b or higher according to the Japanese Society of Nephrology guidelines [29, 30]. However, the findings of our study, which included a long-term observation period of 6 years, suggest that multidisciplinary care can prevent worsening kidney function even in patients with stage 3 CKD.

The present study revealed that the reduction in proteinuria and improvement in ΔeGFR were seen in the DM group over a period of 24 months. Likewise, the improvement in ΔeGFR in the non-DM group was seen over 24 months, but the reduction in proteinuria was evident at just 6 months after starting multidisciplinary care. The rate of nephrosclerosis caused by hypertension in the non-DM group was high, reflecting the aging of the CKD population in Japan. Nephrosclerosis caused by hypertension is characterized by lower proteinuria and a slower decline in eGFR compared with diabetic nephropathy [31]. This was why we found a relationship between the reduction in proteinuria and the improvement in ΔeGFR in the DM group but not in the non-DM group. In addition, no significant difference in ΔeGFR was seen in the stage G3a group over the 24-month period. This may be because of a slower decline in eGFR, fewer or less frequent interventions, or proportionately fewer patients in stage G3a than in other stages. Therefore, the stage G3a group included patients who were not judged by nephrologists to require more intensive treatment via multidisciplinary intervention, since their eGFR values were relatively well preserved.

This study has several limitations. First, it did not include a control group. However, the previously reported meta-analysis found that multidisciplinary care was associated with a lower risk of all-cause mortality in cohort studies but not in randomized controlled trials [19]. Moreover, we could not confirm whether multidisciplinary care contributed to a decrease in the number of patients requiring dialysis. Therefore, further randomized controlled trials and large epidemiological studies that include control groups will be required to confirm the efficacy of multidisciplinary care in patients with CKD. Second, we did not investigate changes in prescriptions, blood pressure, body weight, glycemic control, or laboratory findings other than for kidney function. These factors, which can be influenced by multidisciplinary care, might play an important role in the improvement of both eGFR and proteinuria. It has been reported that the number of medications and prescription patterns among board-certified nephrologists in Japan did not change after the advent of multidisciplinary care [7, 8]. In addition, interventions by registered dieticians and physical therapists were identified as independent predictors of kidney outcomes. However, we could not evaluate what factors contributed most to improving kidney outcomes, such as whether the reduction of salt intake by registered dietitians or exercise therapy by physical therapists lowered blood pressure. Therefore, further investigations are needed to determine the contributing factors of improved adherence to prescription medications, dietary modification, and exercise therapies to prevent the worsening of kidney function. Finally, there may have been some degree of facility and selection bias as a result of variations in the types of health care professionals comprising the multidisciplinary care team and in the educational program between facilities as a result of differences in practice and patient populations. Therefore, educational programs should be standardized to improve the standard of treatment for patients with CKD and an effective and efficient care curriculum should be established.

In conclusion, our findings indicate that multidisciplinary care may significantly slow the decline of eGFR in patients with CKD and be effective regardless of the primary disease. Furthermore, they suggest that multidisciplinary care might be effective even in the earlier stages of CKD. Therefore, multidisciplinary care should be recommended for patients with CKD stage 3–5. Further research is needed to confirm that the CKDE system contributes to improving the standard of medical care for patients with CKD.

## Supplementary Information

Below is the link to the electronic supplementary material.

Supplementary file1 (PDF 115 KB)

Supplementary file2 (PDF 17 KB)

Supplementary file3 (PDF 12 KB)

Supplementary file4 (PDF 13 KB)

Supplementary file5 (PDF 119 KB)

Supplementary file6 (PDF 119 KB)

Supplementary file7 (PDF 128 KB)

Supplementary file8 (PDF 132 KB)

## Data Availability

The data used in this article are available from the corresponding author upon reasonable request.
