# Supplementary material for: Effectiveness and current status of multidisciplinary care for patients with chronic kidney disease in Japan: a nationwide multicenter cohort study
Source: Clin Exp Nephrol. 2023 Mar 31;27(6):528–41. doi: 10.1007/s10157-023-02338-w (PMC10192167; doi:10.1007/s10157-023-02338-w)
Supplement: Supplementary file 1 — Supplementary file1 (PDF 115 KB) [file 10157_2023_2338_MOESM1_ESM.pdf]

Supplementary Figure legends

Supplementary Figure 1. Annual changes in decline of eGFR (ΔeGFR) in the 12 months before and 24 months after initiation of multidisciplinary care in patients with CKD stage 3a (a) and those with 3b (b) at baseline. CKD, chronic kidney disease; eGFR, estimated glomerular filtration rate

Supplementary Figure 2. Kaplan–Meier curves for the incidence of all-cause death in Japanese patients with chronic kidney disease according to DM status. DM, diabetes mellitus

Supplementary Figure 3. Kaplan–Meier curves for the incidence of all-cause death at baseline in Japanese patients with CKD according to disease stage. CKD, chronic kidney disease
