# Supplementary figures and images for: Effectiveness and current status of multidisciplinary care for patients with chronic kidney disease in Japan: a nationwide multicenter cohort study
Source: Clin Exp Nephrol. 2023 Mar 31;27(6):528–41. doi: 10.1007/s10157-023-02338-w (PMC10192167; doi:10.1007/s10157-023-02338-w)

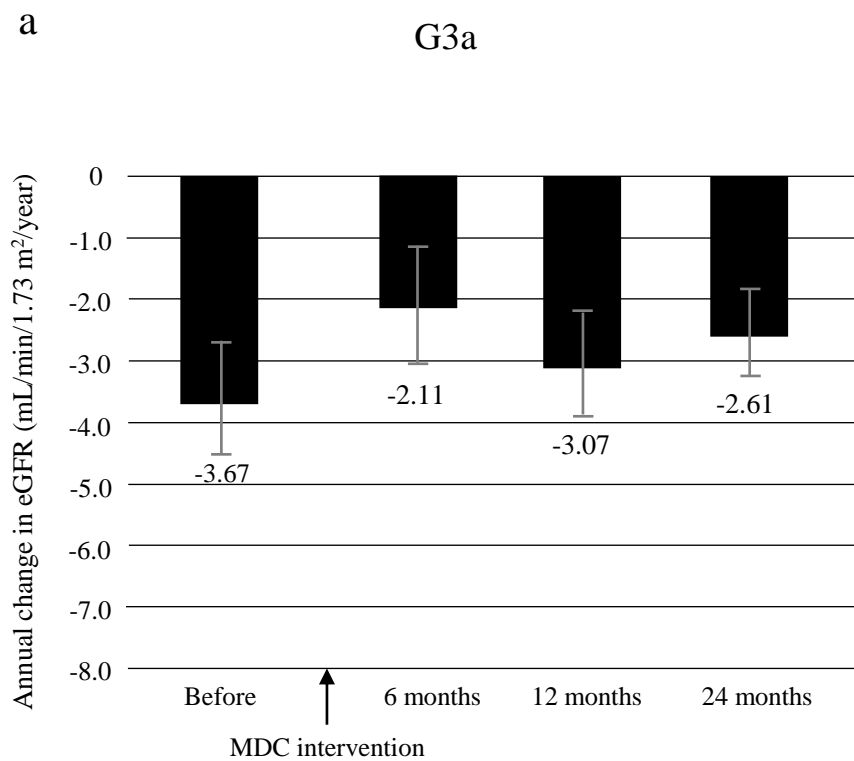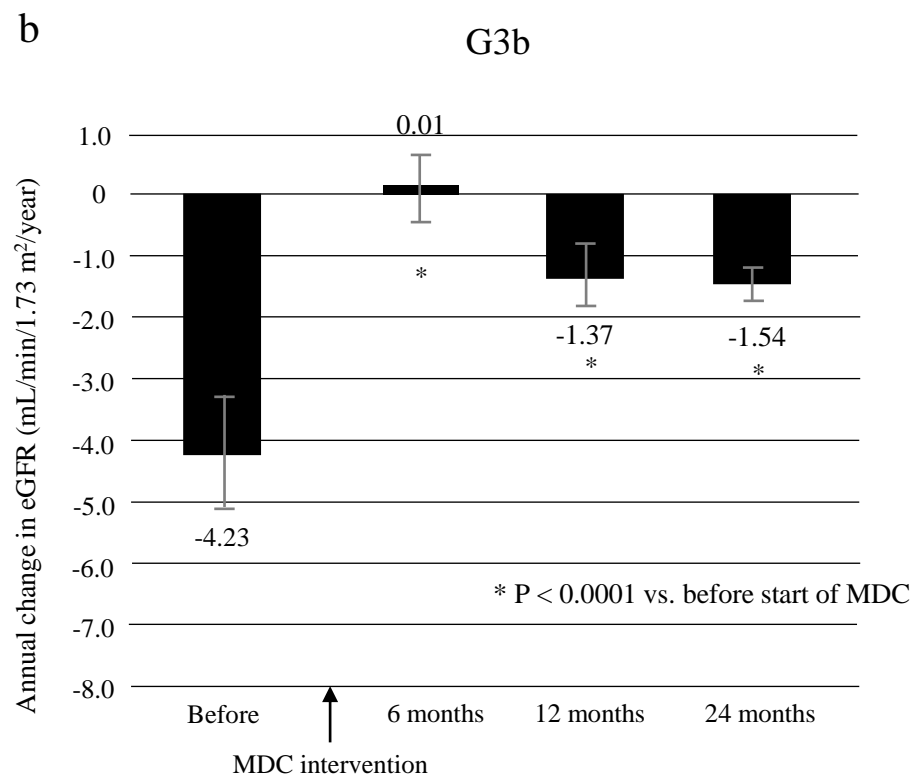

Supplementary Figure 1

Supplement: Supplementary file 2 — Supplementary file2 (PDF 17 KB) [file 10157_2023_2338_MOESM2_ESM.pdf]

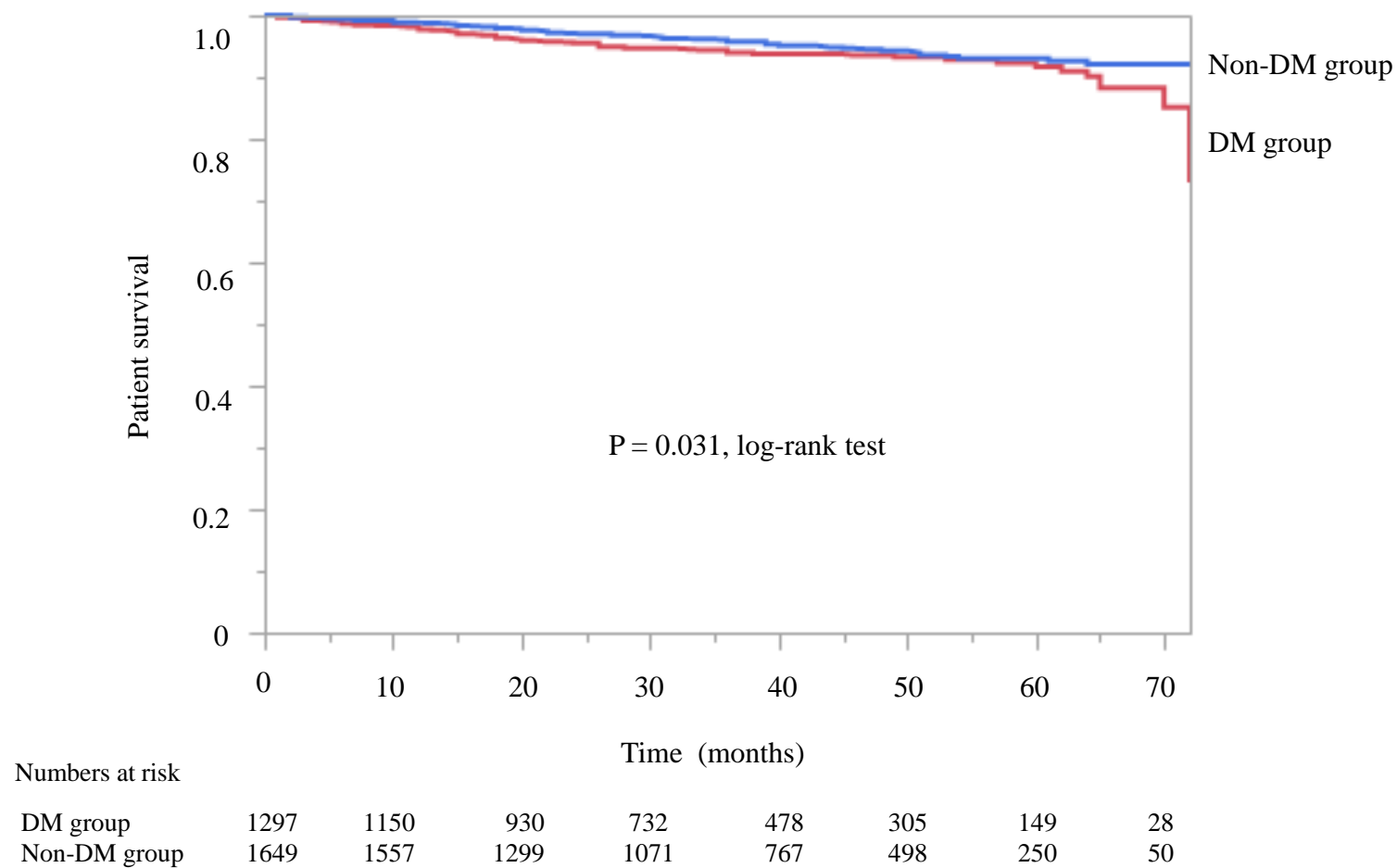

Supplementary Figure 2

Supplement: Supplementary file 3 — Supplementary file3 (PDF 12 KB) [file 10157_2023_2338_MOESM3_ESM.pdf]

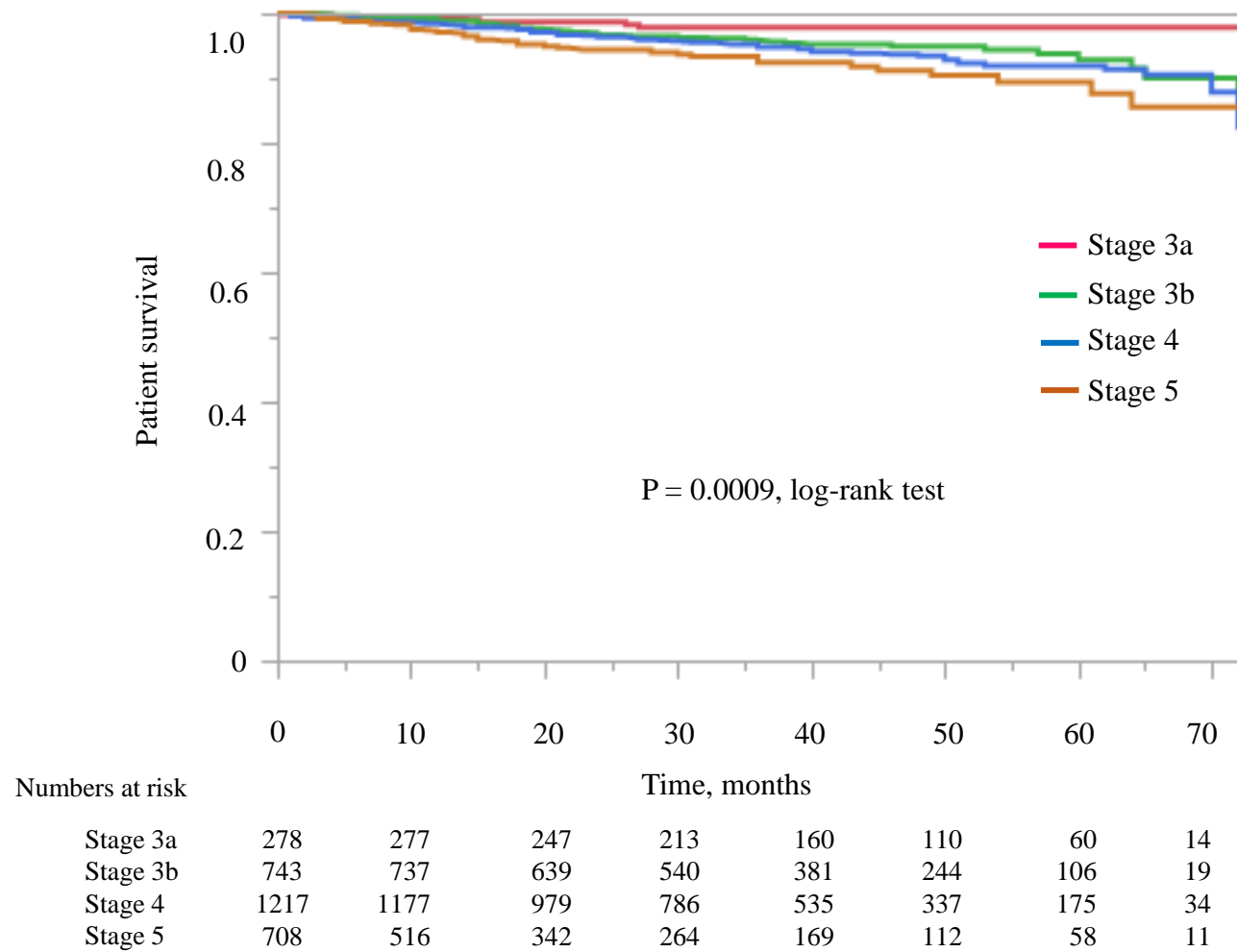

Supplementary Figure 3

Supplement: Supplementary file 4 — Supplementary file4 (PDF 13 KB) [file 10157_2023_2338_MOESM4_ESM.pdf]
