# Supplementary material for: Effectiveness and current status of multidisciplinary care for patients with chronic kidney disease in Japan: a nationwide multicenter cohort study
Source: Clin Exp Nephrol. 2023 Mar 31;27(6):528–41. doi: 10.1007/s10157-023-02338-w (PMC10192167; doi:10.1007/s10157-023-02338-w)
Supplement: Supplementary file 5 — Supplementary file5 (PDF 119 KB) [file 10157_2023_2338_MOESM5_ESM.pdf]

Supplementary Table 1. Spearman's rank correlation coefficients between ΔeGFR and the number of multidisciplinary care team members and the number of interventions by multidisciplinary care team at each time point

Time point	Number of MDC team members		Number of interventions by the MDC team	
	ρ	P value	ρ	P value
6 months	-0.01	0.676	0.04	0.045
12 months	-0.03	0.141	0.05	0.041
24 months	-0.03	0.188	0.08	0.015

MDC, multidisciplinary care.
