# Supplementary material for: Effectiveness and current status of multidisciplinary care for patients with chronic kidney disease in Japan: a nationwide multicenter cohort study
Source: Clin Exp Nephrol. 2023 Mar 31;27(6):528–41. doi: 10.1007/s10157-023-02338-w (PMC10192167; doi:10.1007/s10157-023-02338-w)
Supplement: Supplementary file 7 — Supplementary file7 (PDF 128 KB) [file 10157_2023_2338_MOESM7_ESM.pdf]

Supplementary Table 3. All-cause mortality according to DM status in Cox proportional hazards models adjusted for confounding factors in Japanese patients with chronic kidney disease

Group	Unadjusted			Model 1			Model 2		
	HR	95% CI	P-value	HR	95% CI	P-value	HR	95% CI	P-value
Non-DM	1.00	Reference	-	1.00	Reference	-	1.00	Reference	-
DM	1.42	1.03-1.96	0.031	1.49	1.08-2.06	0.016	1.49	1.01-2.19	0.044

Model 1 was adjusted for basic factors, including age, sex, and history of cardiovascular disease, and model 2 was adjusted in the same way as model 1 but with additional adjustment for body mass index, hemoglobin, serum albumin, estimated glomerular filtration rate, and urinary protein level at baseline. CI, confidence interval; CKD, chronic kidney disease; DM, diabetes mellitus; eGFR, estimated glomerular filtration rate; HR, hazard ratio
