# Supplementary material for: Effectiveness and current status of multidisciplinary care for patients with chronic kidney disease in Japan: a nationwide multicenter cohort study
Source: Clin Exp Nephrol. 2023 Mar 31;27(6):528–41. doi: 10.1007/s10157-023-02338-w (PMC10192167; doi:10.1007/s10157-023-02338-w)
Supplement: Supplementary file 8 — Supplementary file8 (PDF 132 KB) [file 10157_2023_2338_MOESM8_ESM.pdf]

Supplementary Table 4. All-cause mortality according to CKD stage at baseline in Cox proportional hazards models adjusted for confounding factors in Japanese patients with CKD

eGFR and CKD stages	Unadjusted			Model 1			Model 2		
	HR	95%CI	P value	HR	95%CI	P value	HR	95%CI	P value
G3a (eGFR ≥ 45 mL/min/1.73 m^2)	1.00	Reference	-	1.00	Reference	-	1.00	Reference	-
G3b (eGFR, ≥ 30 to 44 mL/min/1.73 $m^{2)}$	2.99	1.29-8.70	0.008	2.42	1.04-7.05	0.038	2.08	0.73-5.98	0.173
G4 (eGFR, 15 to 29 mL/min/1.73 m^2)	3.63	1.62-10.4	0.001	2.49	1.11-7.10	0.025	1.62	0.56-4.68	0.370
G5 (eGFR, <15 mL/min/1.73 m^2)	5.23	2.26-15.0	< 0.0001	3.77	1.61-11.0	0.001	3.02	1.01-9.11	0.048

Model 1 was adjusted for basic factors, including age, sex, history of cardiovascular diseases, and presence or absence of diabetes, and model 2 was adjusted in the same way as model 1 but with additional adjustment for body mass index, hemoglobin, serum albumin, urinary protein to creatinine ratio. CI, confidence interval; CKD, chronic kidney disease; eGFR, estimated glomerular filtration rate; HR, hazard ratio.
